# Between a Clavicle and a Hard Place: Pinch-Off Syndrome in a Subclavian Venous Access Device

**DOI:** 10.51894/001c.144463

**Published:** 2025-09-30

**Authors:** Natalie George

**Affiliations:** 1 College of Osteopathic Medicine Michigan State University

## 82

### INTRODUCTION

Pinch-off syndrome is a rare complication of totally implantable venous access devices (TIVADs) placed via the subclavian vein. Mediports, a type of TIVAD used in chemotherapy, may be affected when the catheter is compressed between the clavicle and first rib, leading to malfunction or fracture. Prompt removal and replacement of broken catheters are essential to ensure uninterrupted chemotherapy. To reduce these risks, physicians should consider using the internal jugular vein for TIVAD placement.

### CASE DESCRIPTION

A 62-year-old female with a history of tobacco use presented with hemoptysis. Further evaluation revealed right-sided lung cancer with bronchial obstruction. Given her presentation, she required Mediport insertion, bronchoscopy, tumor biopsy, and manipulation for bleeding control. On May 13, 2024, a Mediport was placed via the left subclavian vein. Fluoroscopy confirmed proper placement without kinking. The patient tolerated the procedure well. However, in February 2025, she returned due to difficulty flushing the catheter and left-sided chest discomfort during upper extremity movement. A portogram revealed contrast extravasation at the venectomy site, suggesting catheter fracture. Under fluoroscopic guidance, an interventional radiologist removed the fractured catheter and replaced the Mediport via an endovascular approach. The new Mediport was placed in the left internal jugular vein, with final imaging confirming a smooth catheter-to-port connection at the cavoatrial junction.

### DISCUSSION

Mediports are designed for long-term use to maintain consistent treatment regimens. However, the catheter may be compressed and/or fractured in the subclavian vein due to its course between the clavicle and the first rib, resulting in pinch-off syndrome. Although subclavian venous access devices provide rapid access, patient comfort, and optimal mobility, they are more prone to fracture and malfunction than those placed via the internal jugular vein. Our case demonstrates pinch-off syndrome and highlights the advantages of an alternative approach through the internal jugular vein.

### CONCLUSION

The risk of port malfunction in the subclavian vein outweighs its benefits. In such cases, TIVAD placement via the internal jugular vein may be preferable. This case exhibits the importance of procedural planning for patients requiring long-term venous access. Pinch-off syndrome should be considered when planning TIVAD placement to minimize complications.

